# Condensate Formation by Metabolic Enzymes in *Saccharomyces cerevisiae*

**DOI:** 10.3390/microorganisms10020232

**Published:** 2022-01-21

**Authors:** Natsuko Miura

**Affiliations:** Graduate School of Life and Environmental Sciences, Osaka Prefecture University, Sakai 599-8531, Japan; miura.natsuko@biochem.osakafu-u.ac.jp

**Keywords:** *Saccharomyces cerevisiae*, liquid–liquid phase separation, metabolic enzymes, META body, hypoxia

## Abstract

Condensate formation by a group of metabolic enzymes in the cell is an efficient way of regulating cell metabolism through the formation of “membrane-less organelles.” Because of the use of green fluorescent protein (GFP) for investigating protein localization, various enzymes were found to form condensates or filaments in living *Saccharomyces cerevisiae*, mammalian cells, and in other organisms, thereby regulating cell metabolism in the certain status of the cells. Among different environmental stresses, hypoxia triggers the spatial reorganization of many proteins, including more than 20 metabolic enzymes, to form numerous condensates, including “Glycolytic body (G-body)” and “Purinosome.” These individual condensates are collectively named “Metabolic Enzymes Transiently Assembling (META) body”. This review overviews condensate or filament formation by metabolic enzymes in *S. cerevisiae*, focusing on the META body, and recent reports in elucidating regulatory machinery of META body formation.

## 1. Introduction

The intracellular distribution of proteins in cells is strongly related to their function. Protein localization has been extensively studied using budding yeast cells since the development of the GFP-fused protein library in budding yeast cells [1]. First reported in 2003, the library of yeast cells of which fused C-terminus of protein-coding sequences in the genome with GFP (G65T)-coding sequence [2] covers about 75% (4159) of genome-coded proteins in *Saccharomyces cerevisiae* cells (for more details, a haploid strain BY4741 (ATCC 201388), now commercially available). As the localization of most proteins in the library have been recorded and can now be searched in a database (Yeast GFP Fusion Localization Database; https://yeastgfp.yeastgenome.org/ accessed on 13 December 2021), research on protein localization entered a new phase; many proteins exist in locations that vary from those predicted from the amino acid sequence. This is not surprising because proteins, especially metabolic enzymes, exert different functions according to time and occasion, which are called “moonlighting proteins” [3,4]. However, the mechanisms for regulating the intracellular localization of moonlighting proteins are largely unknown. The differential protein localization in cells was sometimes observed depending on the cellular growth [5,6,7,8] or under environmental stress, including nutrient depletion [8,9] and hypoxia [10,11]. There is still a mystery about the biological meanings of spatial reorganization of metabolic enzymes in cells. Some reports have shown the effect of protein condensates to control cellular metabolism [6,10,11]. Here, the recent progress in the protein condensates formed by metabolic enzymes, biological roles of the condensate, and its regulation, mainly focusing on hypoxia-induced protein condensates, were overviewed.

It has now been suggested that metabolic enzymes produce multiple condensates in the cell, and some of these condensates associate with each other in a specific intracellular environment. The term “META body” was first coined in 2021 [12], and the acronym “META” stands for “Metabolic Enzymes Transiently Assembling”. As indicated by the term, the META body is a higher-order enzyme condensate in the cell, comprising condensates formed by glycolytic and purine biosynthesis enzymes that are formed under hypoxic conditions. To discuss the formation, regulation, and function of the enzyme condensates, we first explore the histories associated with the discovery of the unpredicted intracellular localization of enzymes, with emphasis on *S. cerevisiae* cells. These include the formation of filaments by single metabolic enzymes, their regulation, and functions. Then, the condensate formation by a group of metabolic enzymes is examined, focusing on condensates formed by enzymes in the purine synthesis pathway as well as during glycolysis. Finally, the formation of the higher-order enzyme condensate, the META body, and the necessary methodologies and analyses for further research are discussed.

## 2. Filaments Formed by a Single Metabolic Enzyme in *S. cerevisiae*

Before GFP clones were developed, condensate or filament formation by metabolic enzymes in cells has been forecasted by electron microscopy using purified enzymes. The filament formation by some metabolic enzymes was later confirmed in *S. cerevisiae* or other organisms such as *Drosophila* and mammalian cells [13]. For example, acetyl coenzyme A carboxylase (ACC), an enzyme involved in the fatty acid synthesis, purified from animal tissues, formed filaments in electron microscopy [14]. The filament formation of ACC (yeast ortholog, Acc1p) was later confirmed in *S. cerevisiae* cells under prolonged starvation [7,8]. Regulatory mechanisms of ACC filament have been extensively studied on human ACC. The filament formation by ACC is regulated through phosphorylation at serine residue, in addition to adenosine monophosphate (AMP)-activated protein kinase (AMPK) and cAMP-dependent protein kinase (PKA) [15]. Kleinshmidt reported that dissociation of the enzyme filament inactivates the enzyme, and reassembly restored catalytic activity [14]. In vitro studies have indicated that 5 mM citrate concentration induces filament formation of ACC [16]. Although the concentration seemed too high with the plasma concentration of citrate being ~150 μM [17], the intracellular citrate levels of *Escherichia coli* [18] and human breast cancer cells [19] may fall within the range, indicating that the filament formation can be regulated by intracellular metabolites.

Another enzyme, cytidine triphosphate (CTP) synthase, which plays an important role in polynucleotide and lipid synthesis, formed filaments in *E. coli* [20], *S. cerevisiae* [6], *Drosophila* [21], and human-derived HeLa cells [22]. In *S. cerevisiae*, CTP synthase forms filaments under starvation and depends on intracellular pH below 7.0 [23]. The filament formation inactivates CTP synthase [24,25,26] while prolonging the half-life of the enzyme [27]. CTP induces filament formation; in growing bacterial cells, excess CTP possibly induces inactive CTP synthase filaments and control CTP levels in cells [28]. In contrast to bacterial CTP synthase, the polymerization of human CTP synthase increases the enzymatic activity [29]. Furthermore, in *Drosophila* eggs, the polymerization of CTP synthase improves the egg production [30]. These opposite effects of polymerization of CTP synthases among biological species are considered to depend on the three-dimensional conformation of enzymes [30].

In *S. cerevisiae*, an increasing number of metabolic enzymes have been reported to form filaments. Glucokinase (Glk1p) forms filaments in the presence of glucose during the stationary phase, and the polymerization inhibits the enzymatic activity [31]. Glk1p is one of the three enzymes in the hexokinase family. Other members of the hexokinase family, hexokinase 1 (Hxk1p) and 2 (Hxk2p), did not form any filament [31]. Pyruvate kinase (Cdc19p), an ATP-producing enzyme in the glycolytic pathway, also forms molecular condensates or filaments in *S. cerevisiae* under starvation [9,32,33,34], depending on its low-complexity region [35].

Recently, phosphofructokinase (PFK), one of the ATP-consuming enzymes in the glycolytic pathway, formed filaments in breast cancer cells [36] or nerve synapses [37], with unknown mechanisms. These findings imply that the additional enzymes present in *S. cerevisiae* produce filaments in certain environments. The filament formation by a single metabolic enzyme is considered to be a faster technique for regulating the enzymatic activity than protein degradation or de novo protein synthesis, hence conserving cellular energy [38]. While the number of metabolic enzymes that form filaments in cells is increasing, it is not known whether filament formation by a single metabolic enzyme can trigger condensate formation by a group of enzymes that govern the metabolic pathway.

## 3. Predicted Effect of Condensate Formation by Metabolic Enzymes

**Figure 1:**
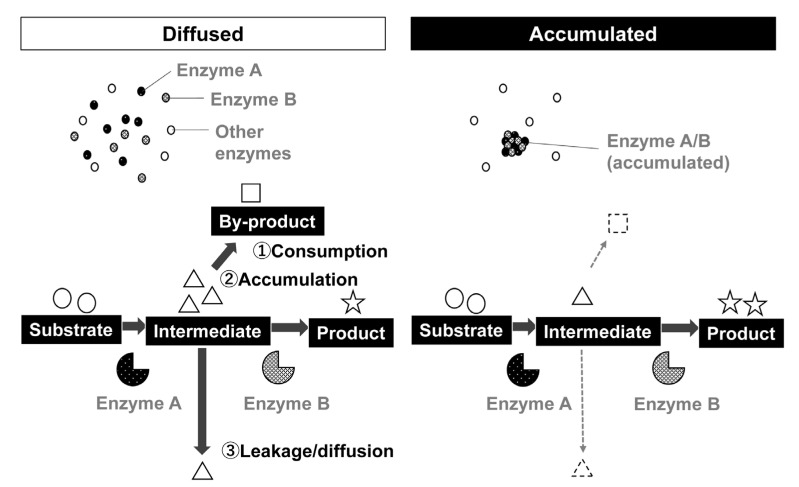
The effect of condensate formation by a group of metabolic enzymes of a metabolic pathway. For continuous enzymatic reactions catalyzed by enzymes A and B, when enzymes A and B are diffused in space (**left**), the intermediate can be consumed by other enzymes or transformed non-enzymatically. The intermediate can also accumulate in a space when enzyme B could not use intermediate immediately. When the reaction is performed in cells, the intermediate can leak from the cells into the extracellular space. As a result, the amount of product becomes smaller than expected. When enzymes are accumulated, the substrate is immediately used for the following reaction catalyzed using enzyme B, thereby efficient production of target products can be achieved. The figure was prepared in reference to Spivey and Merz, 1989 [39]. Copyright© 2020 Japan Society for Bioscience, Biotechnology, and Agrochemistry. https://katosei.jsbba.or.jp/ (accessed on 13 December 2021). DOI number: http://doi.org/10.1271/kagakutoseibutsu.58.10 (accessed on 13 December 2021).

The role of the condensate formation by a group of enzymes that constitutes a metabolic pathway was predicted more than three decades ago. Spivey and Merz have summarized the effect of condensate formation by a group of enzymes in 1989 [39]. According to them, the formation of condensates by metabolic enzymes has various effects, including the smooth transmission of intermediates between enzymes, and avoiding the spread of intermediates or substrates in a space (Figure 1, Adapted with permission from http://doi.org/10.1271/kagakutoseibutsu.58.10 (accessed on 13 December 2021). Copyright©2020 Japan Society for Bioscience, Biotechnology, and Agrochemistry). These effects seem to be related to the concentration of enzymes and substrates in a space. In an attempt to construct artificial metabolic reactions using cells, such as bioethanol production from cellulose, concentrating enzyme cocktail is helpful for efficient manufacture [40]. When using yeast cell surface as a scaffold by cell surface engineering, enzymatic activities of multi-step reactions can be increased. For example, three enzymes, namely endoglucanase, β-glucosidase, and cellobiohydrolase (exo and endo cellulose-degrading enzymes) immobilized on the yeast cell surface exhibited increased glucose production when multiple enzymes were displayed on a single-cell surface [41,42]. When mixing cells displaying only a single type of enzyme each, the productivity is lower than cells displaying enzyme mixture [41]. The effect depends on the concentration of the cells; when cells exist in high concentration (e.g., OD_600_ of 10), displaying a mixed type of enzyme on a single cell seems less effective. This suggests that concentrating a group of enzymes is exerted under low concentrations of the enzymes.

The effect of concentrating enzymes in limited space has also been investigated in vitro. Immobilizing enzymes by binding to a solid surface or encapsulating into hydrogels enhances enzymatic activity, presumably by concentrating the components [43] and stabilizing the three-dimensional folding of enzymes s [44]. For protein condensates using liquid–liquid phase separation, Peeple and Rosen suggested in a preprint that condensate formation increases the cascade reaction activities by SUMOylation enzymes on high K_M_ substrates but not on low K_M_ substrates, with identical k_cat_ [43]. Ura and colleagues reported in a preprint that in vitro condensate formation of lactate oxidase drastically increased k_cat_ and reduced K_M_ [45], suggesting that there is something more occurring other than the effect on substrate channeling by enzyme condensates. This suggests that the enzyme concentration by liquid–liquid phase separation has a somewhat similar effect to the previously known immobilization of the enzyme.

## 4. Condensate Formation by a Group of Enzymes in the Purine Synthesis Pathway

While some enzymes formed filaments or condensates in cells under certain conditions, it was unknown until 2008 whether a group of enzymes that govern some metabolic pathway form condensates to regulate cell metabolism. In 2008, the formation of an enzyme condensate “Purinosome” was reported in human-derived HeLa cells [46]. Purinosome is composed of multiple enzymes in the purine synthesis pathway and is formed in association with microtubules during purine depletion and the G1 phase of the cell cycle [46,47,48,49]. In yeast cells, some enzymes in the purine synthetic pathway form condensates. However, the number of enzymes observed to form condensates varies from mammalian cells, suggesting that there are various mechanisms of purinosome formation between mammalian cells and *S. cerevisiae* cells [5]. Among the purine synthesis pathway enzymes, those located at the branch of the metabolic pathway were found to form protein condensates in *S. cerevisiae* after five days of growth [5]. The effect of the condensate or filament formation by enzymes located at the branch of metabolic pathways on cell metabolism requires further investigation. Some enzymes in the purine biosynthetic pathway, including Ade4p, Ade12p, Ade17p, and Ade5,7p, were shown to form punctate foci in *S. cerevisiae* under nutrient-depleted conditions [9]; the optimization of cell growth conditions might enable the accumulation of all enzymes in the purine biosynthetic pathway in *S. cerevisiae* as well as in mammalian cells.

## 5. Coalesced Metabolic Enzymes under Hypoxia

**Figure 2:**
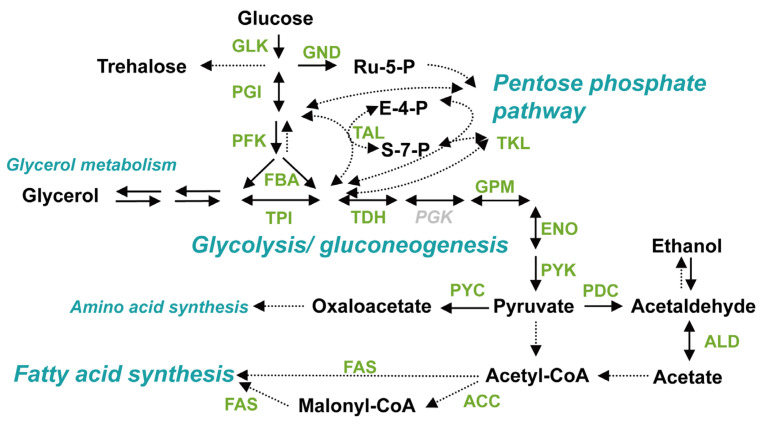
Metabolic enzymes forming condensates under hypoxia in *S. cerevisiae*, determined by fluorescence microscopy. Enzymes found to form condensates under hypoxia by fluorescence microscopic observation using GFP clones [10,11] are illustrated in green characters. Phosphoglucokinase (PGK), a glycolytic enzyme, did not form condensates under hypoxia [11]. GLK: glucokinase, PGI: phosphoglucose isomerase, GND: 6-phosphogluconate dehydrogenase, PFK: phosphofructokinase, TAL: transaldolase, TKL: transketolase, FBA: fructose 1,6-bisphosphate aldolase, TPI: triosephosphate isomerase, TDH: glyceraldehyde-3-phosphate dehydrogenase, GPM: tetrameric phosphoglycerate mutase, ENO: enolase, PYK: pyruvate kinase, PYC: pyruvate carboxylase, PDC: pyruvate decarboxylase, ALD: aldehyde dehydrogenase, ACS: acetyl-coA synthetase, ACC: acetyl-CoA carboxylase, FAS: fatty acid synthetase. In *S. cerevisiae* cells, GLK [31], PYK [9,32,33,34], and ACC [7,8] have been reported to form filaments by themselves.

**Figure 3:**
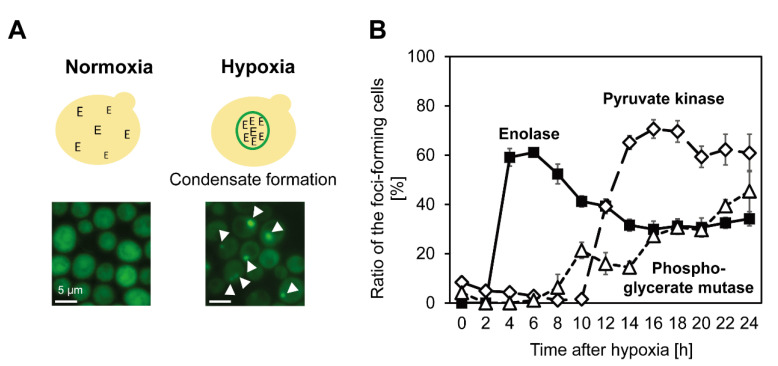
Ordered condensate formation by glycolytic enzymes under hypoxia. (**A**): Condensate formation by enolase (Eno2p) in *S. cerevisiae* under hypoxia. E: enzymes; green: GFP-tagged Eno2p in GFP clones. (**B**): Time-dependent changes in condensate-forming cells under hypoxia, adopted from Yoshimura et al., 2021 [12]. Copyright©2021 International Federation for Cell Biology. https://onlinelibrary.wiley.com/journal/10958355 (accessed on 13 December 2021).

**Figure 4:**
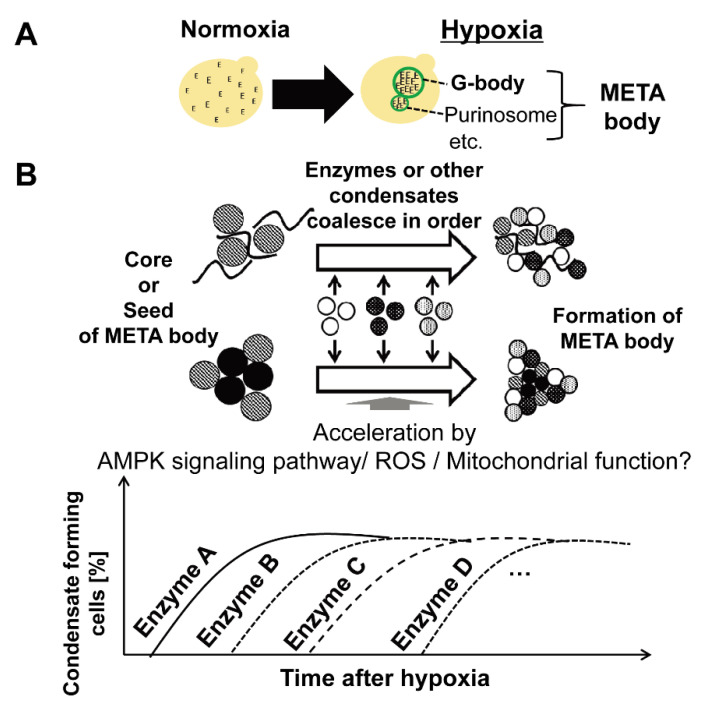
Hypothesis for the formation of Metabolic Enzymes Transiently Assembling (META) body. (**A**) The formation of META body under hypoxia. Proteins, including metabolic enzymes and translation-related proteins, form condensates individually named as G-body or purinosome. META body includes both molecular condensates. These protein condensates formed under hypoxia are collectively named META body [12]. E: metabolic enzymes; green circle: condensates. (**B**) A hypothesis for META body formation. META body formation is presumably regulated through AMPK signaling, ROS, and mitochondrial function, as previously reported [11]. Enzymes or several condensates coalesce to form the META body. Closed circle: proteins; waved line: biomolecules such as polynucleotides.

In 2013, another group of metabolic enzymes governing the glycolytic pathway were found to form large-scale molecular condensates in *S. cerevisiae* under hypoxia [11]. The existence of the condensate in cells under hypoxia was reconfirmed in 2017, and the condensate was named “Glycolytic body” or “G-body” [10]. Moreover, similar condensate was also observed in mammalian cells, and the condensate in mammalian cells was named “G-body” [10] or “Glucosome” [50]. The number of G-body-associated proteins identified through pull-down assay followed by liquid chromatography and mass spectrometry (LC-MS) was more than 100, and microscopic observations using GFP clones showed that most glycolytic enzymes coalesce under hypoxia in a similar localization in the cell (Figure 2) [10,11]. While the machinery of G-body formation is still unclear, it was suggested that enzymes that form molecular condensate under hypoxia coalesce in order (Figure 3, Adapted with permission from ref. [12]. Copyright©2021 International Federation for Cell Biology. https://onlinelibrary.wiley.com/journal/10958355 (accessed on 13 December 2021) [12], suggesting the existence of coordinated regulatory mechanisms of the condensate formation (Figure 4). Not only glycolytic enzymes, but also Ade57p—a member of purinosome—were specifically immunoprecipitated with condensate-forming enolase [11], suggesting that enzymes of other metabolic pathways, or other types of condensates, are also involved in the condensate formed by glycolytic enzymes. Indeed, it was later reported that enzymes of the purine synthetic pathway that forms purinosome also form molecular condensate in cells under hypoxia [51]. However, the effect of synthetic purine enzymes assembled under hypoxia is unspecified. The large-scale protein assembly in cells formed under hypoxia, “META body” [12], is now presenting novel cellular machinery that potentially newly regulates cell metabolism. In this review, protein condensates formed under hypoxia are collectively referred to as the “META body”.

## 6. Effect on Cellular Metabolism by Spatial Reorganization of Metabolic Enzymes under Hypoxia

The effect of META body formation under hypoxia remains unclear. Some experimental results support the hypothesis that META body formation accelerates cell metabolism; more specifically, glycolysis.

Unlike other metabolic enzymes that form molecular condensates, yeast enolase (Eno2p), which is an ortholog of human ENO1 (enolase that is expressed whole body), and the homolog of Eno2p, Eno1p, are identified to have amino acid residue, which is critical to condensate formation [11]. Using the single-amino-acid substituted mutant (ENO2V22A), it was shown that metabolic turnover of ^13^C-labeled glucose to pyruvate and oxaloacetate is upregulated in cells with condensates under hypoxia, compared with cells without condensates or under normoxia [11], suggesting the role of condensates formed by enolase in increasing glucose turnover under hypoxia.

Amino-acid dependency on the condensate formation by PFK, a component of molecular condensate under hypoxia, has also been investigated. PFK has a predicted intrinsically disordered region (IDR) with 140–165 amino acids near the N-terminus, and deletion of the IDR inhibits condensate formation by PFK [10]. The proliferation of yeast daughter cells under hypoxia reduced in PFK-deficient cells [10]. Whether the deletion of IDR of PFK affects cell metabolism under hypoxia requires further investigation.

The above two reports focused on the condensate formation by a single enzyme component of the META body. They could not determine the effect of a body of molecular condensate by different enzymes. It is important to elucidate the effect of large-scale enzyme assembly on cell physiology, including metabolism, methods to control the association and dissociation of condensates by metabolic enzymes. Moreover, there is a need to determine the kinetics of each enzyme in the condensate, as enzyme kinetics in condensates can change drastically [45] and can affect parameters such as those used for metabolic flux analysis. These would provide the apparent activity of condensed enzymes at bulk concentrations, thereby estimating the effect on the whole cell. To determine the absolute effect of enzyme-induced condensate formation, the local concentration of enzymes and substrates should be investigated. To experimentally determine the effect of condensate formation in the cells, it would be useful to develop a novel method for reconstructing the condensates formed by metabolic enzymes in vitro, preferably using only biomolecules found in the cells.

## 7. Regulatory Machinery of Condensate Formation under Hypoxia

Attempts to reveal the regulatory machinery of the META body have mainly been performed using knockout strains and chemical inhibitors. Jin and colleagues have screened the yeast gene knockout library and found that genes, including *SNF1*, an ortholog of human AMP kinase that governs one of the signaling pathways, affect condensate formation by PFK [10]. Knockout of *SNF1* also affects the condensate formation by enolase [11] and it was indicated that knockout of *SNF1* does not completely inhibit condensate formation but delays it [10,12]. *SNF1* deletion mutant grows slower compared to wild type. However, by using an AMPK inhibitor, dorsomorphin, that has no significant effect on cellular growth, it was determined that involvement of *SNF1*/AMPK signaling pathway in the condensate formation was not through growth inhibition [12]. The effect of inhibiting META body formation was reversed using a nonspecific AMPK activator, metformin [12], suggesting that chemicals may reversibly control the formation and dissociation of the META body.

Inhibition of the formation of condensates through many chemicals has revealed the contribution of de novo protein synthesis, mitochondrial function, and reactive oxygen species (ROS) in spatial reorganization of enolase under hypoxia, besides the AMPK signaling pathway [11]. It seems controversial that mitochondria, which are thought to be “silent” under hypoxia, are incorporated in regulating metabolic enzymes. However, recent metabolomics analysis has shown that the metabolic pathway in mitochondria is operational in mammalian cells [52] and *S. cerevisiae* [53], even under hypoxia. Further investigation is needed to connect the “dot” to elucidate the mechanisms of META body formation.

Recently, the existence of RNA in the formation of protein condensates through yeast PFK (Pfk1p), enolase (Eno1p), and fructose 1,6-bisphosphate aldolase (Fba1p) has been reported [54]. Many RNA-binding proteins have been found in the META body, and it is possible that certain RNA molecules are the key to META body formation in the cell. mRNA sequences that associate with the three enzymes under normoxia have reached 1540 in total, and 131 are recognized by all three enzymes, suggesting that some RNA molecules can bind to META body-forming enzymes simultaneously [54]. Recently, Morales-Polanco and colleagues reported that mRNAs of glycolytic enzymes form condensates in cells [55], exhibiting spatial organization of mRNAs of META body-forming enzymes. Whether mRNA localization affects protein localization under hypoxia to affect META body formation requires further investigation.

While some have been suggested by the current progress in the field, the regulatory mechanisms of protein condensates remain unclear. When using chemicals, it is inevitable that the chemical might possess side effects for non-targets in the cell. Using additional chemicals that counteract the first chemical [12] might be effective in elucidating the chemical effect of the condensates. Gene knockout also has a similar problem; it is recommended to conduct a compensate experiment by expressing the deleted gene in the cell. When incorporating certain molecules into protein condensates, the side effects of the molecule should be considered because bulky molecules can interfere with the molecular interactions inside condensates. Finally, the systems to achieve hypoxia should be carefully selected, since some methods widely used for hypoxic culture are unsuitable for observation of the META body, especially for a long time [12]. Overcoming these problems would enable the elucidation of the regulatory machinery of protein condensates under hypoxia.

## 8. Summary and Future Perspectives

In this decade, different metabolic enzymes are incorporated into a cellular “membrane-less organelle” possibly by liquid–liquid phase separation, phenomena that have long been predicted. *S. cerevisiae* was the center for the discovery, especially for the META body, as different GFP-tagged protein libraries were obtained [1] and widely used by various scientists. The formation of molecular condensate did not always occur in a “normal” situation; for example, in cells with a rich energy source and oxygen supply. The condensate formation has been observed in a nutrient-limited or stressed situation in cells, proposing the role of condensates by metabolic enzymes in cell proliferation [10]. Alternatively, standard cell culture methods might be too “rich” for cells to exert cellular qualities to respond to environmental stress, survive or compete to leave offspring to the next generation. These findings may provide implications for our views on the role of cell metabolism in nature.

The term “aggregate” is often applied to inactivated cellular components due to protein overexpression or inevitable cellular processes [56,57]. Here, a question arises whether the condensate or filament formation by metabolic enzymes is a passive or active process in the cells. Most META body-containing metabolic enzymes belong to the glycolytic pathway, which is a central metabolic pathway conserved over various species. Loss of metabolic function by aggregation of central metabolic enzymes leads to the death of the organism. Therefore, the “passive” formation of inactive aggregates should be avoided during evolution. It seems more relevant to believe that the property of central metabolic enzymes to form condensates under hypoxia has been positively selected during evolution, especially in environments where hypoxia is prevalent. It is suggested that the condensate-forming property of the N-terminal region of enolase is conserved over species, despite some variations between homologs [11]. In terms of defending the cells against the stress of inevitable aggregation, various machineries seem to exist, including the formation of stress granules [58] and their clearance by autophagy [59,60], in addition to unnecessary protein degradation by proteasome [61]; these also form phase-separated compartments in the cells. The relationships between these protective machineries and the condensates formed by metabolic enzymes are unknown; however, these mechanisms may be used to remove the metabolic condensates once they are no longer needed.

There might be a strict difference between the regulation of enzyme catalytic activity by filament formation and the acceleration of metabolic turnover due to condensate formation. Filaments formed by metabolic enzymes have a limited number of enzymes, whereas condensates, such as the META body, are organized by various proteins, including metabolic enzymes and translational factors. The filament formation by metabolic enzymes is used in regulatory functions of a single enzyme, such as the activation of ACC [14] and the inactivation of bacterial CTP synthase [24,25,26]. On the other hand, condensate formation appears to upregulate the metabolic pathway [10,11,46]. Condensate formation, unlike filamentous enzymes, may not contribute to direct substrate channeling between enzymes. Rather, condensate formation may aid in the enzymatic cascade reactions by inhibiting the diffusion of substrates, reactive intermediates, and cofactors.

While different enzymes form condensates such as the META body, the regulatory machineries are still not elucidated. Developing tools that can control the association and dissociation of different enzymes in cells would in turn show the principle of condensate formation in the cell. When investigating the effect of condensate formation on enzymatic activity, methods for investigating cell metabolism should be carefully selected or newly developed, considering the effect of changes in the catalytic activity of enzymes in condensates. When investigating large-scale molecular condensates, the timing for investigation should also be carefully set, as the incorporation of each metabolic enzyme into the META body is tightly regulated over time [12]. Note that there is a possibility that these protein condensates are formed by numerous small protein condensates with a limited number of enzymes, as Menard and colleagues discussed metabolic compartments in human muscle [62]. Some enzymes found in the META body, PFK [36], PYK [9,34], and ACC [7,8], are also reported to form filaments in growing mammalian or yeast cells, and the relationship between filaments by a single enzyme and condensates by many enzymes require further investigation. Developing these tools and methods would accelerate the investigation of enzymatic condensates in cells.

The preservation of protein condensates by various species of metabolic enzymes also provides insight into the role of protein condensates in cells. Some condensates, such as purinosome [46] or condensate by a glycolytic enzyme glyceraldehyde-3-phosphate dehydrogenase [63], were originally reported in mammalian cells. Alternatively, the formation of the META body, or condensates by glycolytic enzymes, were first found in *S, cerevisiae* [11], and later confirmed in mammalian cells [10,50] and synapses of *C. elegans* [37]. In studies using mammalian cells, cancer cell lines are used. Further investigation is required to find which types of cancer cells form the META body. It would also be essential to study normal tissue in the body, especially for investigating META body, as some tissues (cartilage, lungs, heart, brain) or cells, such as immune and stem cells are exposed to some degree of hypoxia in some cases. Moreover, differences and similarities of protein condensates between META bodies in eukaryotes and prokaryotes or plant cells might give us more insight into the use of metabolic proteins to sustain the life of organisms. Such investigations in turn would give us more insights into the necessary characteristics of metabolic enzymes that sustain cellular energy sources even under different environmental changes.
